# Facile Synthesis of Porous Silicon Nanofibers by Magnesium Reduction for Application in Lithium Ion Batteries

**DOI:** 10.1186/s11671-015-1132-8

**Published:** 2015-10-28

**Authors:** Daehwan Cho, Moonkyoung Kim, Jeonghyun Hwang, Jay Hoon Park, Yong Lak Joo, Youngjin Jeong

**Affiliations:** School of Chemical and Biomolecular Engineering, Cornell University, Ithaca, NY 14853 USA; School of Electrical and Computer Engineering, Cornell University, Ithaca, NY 14853 USA; Department of Chemical Engineering, Massachusetts Institute of Technology, Cambridge, MA 02139 USA; Department of Organic Materials and Fiber Engineering, Soongsil University, Seoul, 156-743 Korea

**Keywords:** Porous silicon nanofibers, Electrospinning, Magnesium reduction, Graphene, Lithium ion batteries

## Abstract

We report a facile fabrication of porous silicon nanofibers by a simple three-stage procedure. Polymer/silicon precursor composite nanofibers are first fabricated by electrospinning, a water-based spinning dope, which undergoes subsequent heat treatment and then reduction using magnesium to be converted into porous silicon nanofibers. The porous silicon nanofibers are coated with a graphene by using a plasma-enhanced chemical vapor deposition for use as an anode material of lithium ion batteries. The porous silicon nanofibers can be mass-produced by a simple and solvent-free method, which uses an environmental-friendly polymer solution. The graphene-coated silicon nanofibers show an improved cycling performance of a capacity retention than the pure silicon nanofibers due to the suppression of the volume change and the increase of electric conductivity by the graphene.

**Electronic supplementary material:**

The online version of this article (doi:10.1186/s11671-015-1132-8) contains supplementary material, which is available to authorized users.

## Background

Rechargeable lithium (Li) ion batteries have been widely used in small scale electronics such as smartphones and laptops to date [1, 2]. Currently, they are one of the most popular types of batteries used in portable electronics. The ever-expanding demand for these batteries has led to their recent applications with plug-in hybrid/electric vehicles and energy storage systems. Evidently, there has been rapidly growing efforts to develop new electrode materials with higher energy/power density and longer cycle life at lower costs [3, 4]. Commercially, graphite has been used as an anode material for Li-ion batteries because of its flat working potential, high coulombic efficiency, and good cycleability [5, 6]. However, it has a significantly low theoretical capacity of 370 mAh/g. Over the last decade, Li-ion batteries have been extensively studied to improve the performance of their battery components (anode/cathode material, electrolyte, and separator) [7, 8].

Recently, some materials such as silicon (Si), tin, and germanium have been studied for their use as anode electrodes of Li-ion batteries because of their high theoretical capacities [9]. Among the potential material candidates, a Si-based material has attracted considerable interest because of its highest-known theoretical charge capacity of 4200 mAh/g at 415 °C or 3572 mAh/g at room temperature as a form of Li_22_Si_5_ or Li_15_Si_4_, respectively [10–13]. Moreover, a Si is one of the most common elements on earth, which leads to cost-effective production. However, it is well known that a huge volume change (>400 %) is vassociated with the lithium insertion and extraction in Si anodes due to the large amount of Li-ion uptake in Si [14]. The volume changes induce stresses on Si anodes and then cause their pulverization, which eventually makes loss of electrical contact and final capacity fading during cycles [15, 16]. This problem is a significant challenge to overcome for a commercialization of Si anodes in battery application.

Recently, most efforts have been made to improve the cycling performance of Si anodes by encapsulation or Si nanocomposition. The most facile approach is to use a buffering matrix encapsulating Si nanoparticles. The matrix plays a role as a buffer to absorb the severe volume change of Si, which may suppress its pulverization [17–19]. It has been reported that Si/carbon composites showed much better cycling performance than a pristine Si material [20, 21]. In addition, other approaches have shown that the cycling performance of Li-ion batteries can be improved by the tuned Si anode materials such as nanowires and hollow structures [22, 23]. Even though the modified Si materials have been successfully fabricated, the processing methods are often extremely complicated and do not lend themselves well to a low-cost high-throughput scale-up.

## Methods

### Materials

Polyvinyl alcohol with a molecular weight of 78,000 was purchased from Polysciences, Inc (Warrington, PA). The polymer is 99.7 % hydrolyzed so that it has nearly the same number of corresponding hydroxyl groups as the degree of polymerization. A Si tetraacetate precursor and nonionic surfactant of Triton X-100 (p-tertiary-octylphenoxy polyethyl alcohol) were purchased from Sigma-Aldrich Company. Tap water was used as a solvent to dissolve the polyvinyl alcohol (PVA) polymers and to disperse the Si tetraacetate precursor.

### Fabrication of PVA/Si Precursor NFs

PVA/Si precursor solution was prepared by mixing both PVA and Si precursor aqueous solutions. At first, the Si tetraacetate precursors were put in water/acetic acid (7/3 wt.%) and stirred on an ice bath for around 2 h in order to prevent the aggregation of the Si precursors (Additional file 1: Figure S1). To make 10 wt.% of a PVA solution, the PVA polymer was added to water and put into a 95 °C oven for 6 h. After the PVA solution was cooled to room temperature, the two prepared solutions were mixed and stirred for 2 h. A few drops (0.5 wt.% to water) of X-100 surfactant were added to the mixture solutions, followed by stirring them for a couple of minutes.

A 5-mL plastic syringe with a 20-gauge needle was loaded with the prepared spinning dope. A high-voltage power supply (gamma) applied a positive charge to the needle. To collect the PVA/Si tetraacetate composite nanofibers (NFs) in this study, a metal plate was used as a collector that was grounded. A micropump (Harvard Apparatus, Holliston, MA) was used to infuse the solution and to eject it toward to the collector. An 18 kV voltage was maintained at the needle tip. The distance between the collector and the needle tip was set at 15 cm, and a constant flow rate of solution was set to 1.2 mL/h.

### Formation of Porous Si NFs

The as-spun NFs were placed in an air box furnace to calcine them at 600 °C and then held for 1 h, which removed the PVA polymers and formed silica (SiO_2_) NFs. The SiO_2_ NFs and Magnesium (Mg) powders were placed together at the opposite ends in a ceramic boat and then sealed by using a cylindrical homemade container that has a small hole at each end. The molar ratio SiO_2_ to Mg was approximately 1 to 2.5. The samples were put into a vacuum tube furnace (MTI, OTF-1500X-UL-4) and heat-treated at 650 °C for 30 min to reduce the SiO_2_ NFs. While being held at 650 °C, the vacuum pressure was kept on the magnitude of 10^−4^ Torr. The Mg-treated NFs were immersed in 1 M HCl solution for 4 h to dissolve the magnesia and then washed using water to obtain the porous Si NFs, followed by drying for 6 h in a vacuum oven.

A plasma-enhanced chemical vapor deposition (PECVD) method was utilized to cover most surface of the porous Si NFs with a graphene material. The samples were treated in a N_2_ ambient for 10 min. Then, the mixture of CH_4_ and H_2_ with a ratio of 2:1 was introduced with 140 W of radio-frequency (RF) power for the graphene layer on the samples (Additional file 1: Figure S2).

### Characterization and Electrochemical Analysis of the Si NFs

The morphology of NFs at each step was evaluated with a Leica 440 scanning electron microscope (SEM) after being coated with Au-Pd. X-ray diffraction (XRD) measurements were performed to investigate the crystal structures using a Scintag Theta-theta X-ray Diffractometer (nickel-filtered CuKa radiation, *λ* = 1.54 Å) operating at 45 kV. All the data were collected in the 2*θ* range of 15–70° with a step of 0.03° and a scanning rate of 5°/min. Energy dispersive X-ray (EDX) analysis was used in conjunction with focused ion beam SEM. The energy of the beam was 20 keV and 0.58 mA where the targeting area of the beam was approximately 1 μm^3^. The internal structure of the specimens was observed by using a transmission electron microscope (TEM). The TEM images were taken using a Tecnai T-12 at an accelerating voltage of 120 kV.

To prepare a graphene-coated Si NFs electrode, the active materials were blended with a Super P (Timcal) and poly(acrylic acid) (Mw = 3,000,000, Aldrich) to prepare a homogenous slurry according to the ratio 70:15:15 (*w*/*w*/*w*), in *N*-methyl-2-pyrrolidinone (NMP, Aldrich). To form a working electrode, the prepared slurry was drop-cast on a copper current collector and then dried to completely evaporate NMP in a vacuum oven at 80 °C for 4 h. A Li metal was used as a counter electrode, and Celgard 2500 (polypropylene) was inserted as a separator between the working electrode and the Li metal chip. We used a homemade electrolyte that composed of 1 M LiPF_6_ in a solution of ethylene carbonate and fluoroethylene carbonate (1:1 *w*/*w*). A 2032-type coin cell was assembled in an Ar-filled glove box. After fabricating electrodes using the prepared materials, galvanostatic charge/discharge tests of a coin battery cell were performed by using charge/discharge cyclers (MTI) for cycling performance, which was carried out within a cutoff voltage window from 0.01 to 2.0 V versus Li/Li^+^. The structural changes of the active electrodes were investigated by disassembling the cells in an Ar-filled glove box before cycling and after 50 cycles. The specific capacity was calculated on the total electrode weight.

## Results and Discussion

Herein, highly porous Si NFs were fabricated by a low-cost electrospinning method using a water-based spinning dope, followed by a heat treatment and a Mg reduction. Electrospinning is a technique by which fibers, with diameters ranging from micrometers to a few nanometers, can be produced from electrically driven jet of polymeric fluid [24]. The porous Si NFs are coated with a graphene by using a PECVD for use as an anode material of Li-ion batteries.

A chemical vapor deposition (CVD) is one of the promising methods to synthesize large area of high quality graphene by applying the mixtures of hydrogen and methane fed into a hot-wall reactor for chemical reactions. However, it takes a long process due to slow increase temperature. It has a critical problem in which the applied high temperature causes substrate evaporation by a catalytic reaction with methane [25]. To improve the processability in this study, a PECVD has been applied to form graphene on Si NFs using H_2_ and CH_4_ at around 400 °C temperature (see Additional file 1: Figure S2). It has a relatively lower quality compared to the film formed by CVD in high temperatures, but the quality of graphene/graphitic film for anode application is not critical as much as that of other applications such as RF devices, transistors, and sensors. In addition, it is broadly applied due to its uniformity in large area, relatively low process temperature (400 °C), easy operations, and simple configurations [26].

**Fig. 1:**
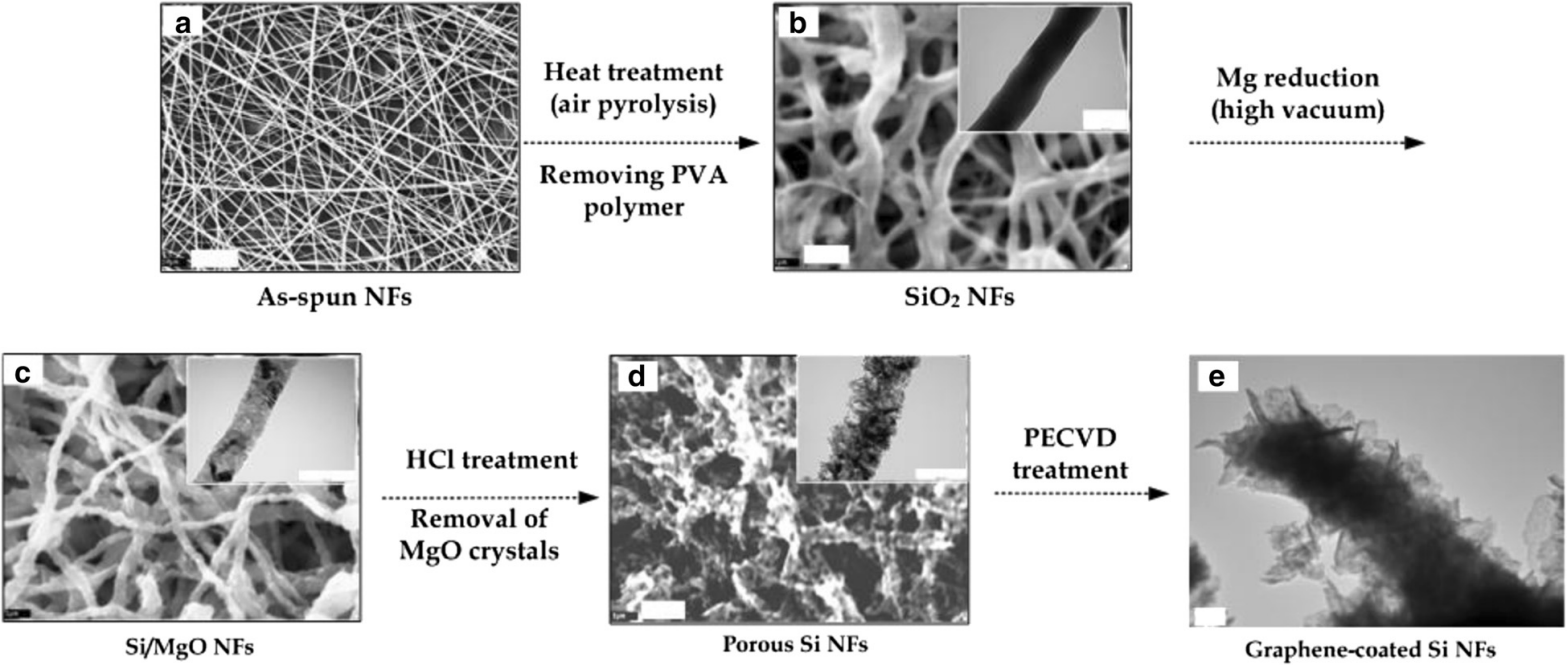
Overall fabrication scheme of porous graphene-coated Si NFs: SEM images (**a**–**d**) and TEM image (**e**) of the fabricated NFs at each step (the scale bars of (**a**), (**b**–**d**), and (**e**) represent 10, 1, and 100 nm, respectively. TEM images of *insets* (**b**–**c**) and (**d**) have the scale bars of 100 and 200 nm)

In this study, the porous Si NFs can be mass-produced by a simple and solvent-free method, which uses an environmental-friendly polymer solution. The overall process to form the porous graphene-covered Si NFs is illustrated in Fig. 1. As can be seen in Fig. 1a, the uniform as-spun PAV/Si tetraacetate composite NFs were fabricated by electrospinning method and showed good fiber morphology having a diameter ranging from 250 to 500 nm. The electrospinning dope was prepared by incorporating high content of Si precursors in a PVA polymer solution, higher than 50 wt.% to the PVA polymer, which was a stable and homogenous mixture solution by suppressing a reaction of the Si tetraacetate precursors by using a cold bath and an excess of acetate in the solution (see Additional file 1: Figure S1). A schematic illustrates the interaction between PVA and Si tetraacetate in the spinning dope (see Additional file 1: Figure S3). Strong hydrogen bonding between the acetate group of Si precursor and the hydroxyl group of PVA chains enables the high loading of Si precursor into PVA polymer solutions. The FTIR spectroscopy showed the bonding between two materials (see Additional file 1: Figure S4).

**Fig. 2:**
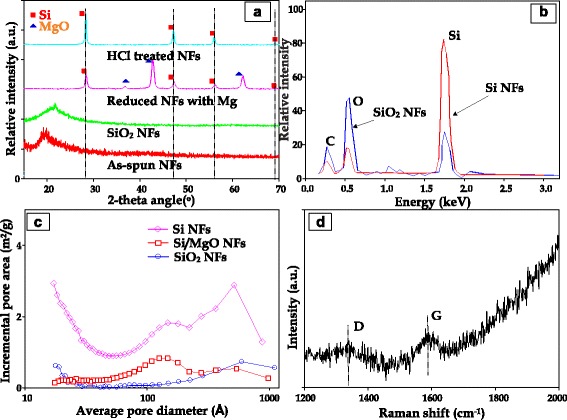
**a** XRD profiles of the fabricated NFs at each step, **b** EDX analyses of SiO_2_ NFs and HCl-treated NFs, **c** incremental pore areas of each fiber, and **d** Raman spectrum of graphene-coated Si NFs

To decompose the organic components in the as-spun NFs and then generate the SiO_2_ NFs, they were pyrolyzed at 600 °C in an air furnace for 1 h. After removing the PVA polymer from the as-spun PVA/Si precursor NFs, the continuous SiO_2_ NFs were formed. It should be noted that there is a strong hydrogen bonding between the acetate groups of Si precursors and the hydroxyl groups of PVA chains in the spinning dope [27]. The bonding enables the Si precursors to bind to the hydroxyl groups in the PVA backbone chains and develop SiO_2_-networked NFs by condensing the acetate groups between the adjacent Si precursors during the heat treatment at 600 °C in air. The SEM image in Fig. 1b shows the fiber morphology of the continuously formed SiO_2_ NFs. Most residual carbon is removed by thermal decomposition of PVA during its calcination step. The TEM image in an inset of Fig. 1b and the broad peak at 2*θ* = 23° of the XRD profile in Fig. 2a revealed that the formed SiO_2_ NFs had the amorphous structures (see Additional file 1: Figure S5a).

**Fig. 3:**
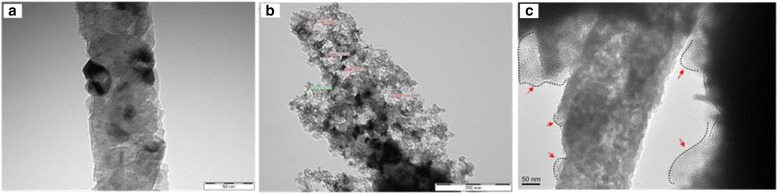
TEM images of Mg-treated (**a**), HCl-treated (**b**), and PECVD-treated NFs (**c**) (the scale bars of images (**a**, **c**) and (**b**) indicate 50 and 200 nm, respectively)

As shown in Fig. 1c, the SiO_2_ NFs were reduced by a gaseous Mg treatment and then converted into continuous crystalized NFs containing both Si and magnesia (MgO) crystals as in a reaction shown in Eq. 1. The small (approximately 10 nm size) and discrete crystalline domains in the generated NFs were evenly distributed throughout the fiber (see Fig. 3a and Additional file 1: Figure S5b). The presence of both MgO and Si was clearly confirmed by the characteristic XRD peaks in Fig. 2a. The silica reduction via Mg vapor was operated at a much lower temperature (650 °C) and shorter duration (30 min) than other methods such as the carbothermal reduction (>1600 °C) or the electrochemical reduction [28].

1 $$ \mathrm{S}\mathrm{i}{\mathrm{O}}_2\left(\mathrm{s}\right) + 2\mathrm{Mg}\left(\mathrm{g}\right)\to \mathrm{S}\mathrm{i}\left(\mathrm{s}\right) + 2\mathrm{M}\mathrm{g}\mathrm{O}\left(\mathrm{s}\right) $$

2 $$ \mathrm{M}\mathrm{g}\mathrm{O}\left(\mathrm{s}\right) + 2\mathrm{H}\mathrm{C}\mathrm{l}\left(\mathrm{l}\right)\to \mathrm{M}\mathrm{g}\mathrm{C}{\mathrm{l}}_2\left(\mathrm{a}\mathrm{q}\right) + {\mathrm{H}}_2\mathrm{O}\left(\mathrm{l}\right) $$

The porous Si NFs were obtained by immersing the MgO/Si NFs in a 1 M HCl solution for 4 h, followed by washing the MgO crystals off using water. Equation 2 indicates that the MgCl_2_ might be recycled to obtain Mg via electrolysis. As can be seen in Fig. 2a, the typical XRD patterns exhibited that the MgO crystals were removed, which is in a good agreement with those of Si and MgO in the results studied by Bao et al. [29].

In Fig. 2, the SiO_2_ NFs showed much higher peak of oxygen and smaller Si peak than the HCl-washed NFs. After treating the SiO_2_ NFs with Mg, the SiO_2_ materials were reduced by forming Si/MgO crystals. It was noted that the process of HCl washing removed the MgO crystals in the NFs but did not completely dissolve the carbon and remove the oxygen. Meanwhile, it is worthwhile to note that a modest oxygen peak was clearly observed in the EDX pattern of Fig. 2b. We believe that the residual carbon was originated from the organic polymer and the oxygen originated from a bind between water in a dilute HCl solution and Si, while the Si/MgO composite NFs were being treated during the HCl-washing step. However, it would be very difficult to discern the oxygen source in the Si NFs. It suggested that small amount of oxygen might be originated from the SiO_2_ residue that did not react with Mg during the reduction process. It should be noted that our study does not involve a toxic hydrofluoric (HF) solution treatment step unlike a reference paper [28]. The HF treatment process poses high health and safety hazards; hence, it is difficult to adapt into a scale-up procedure.

An observation in Figs. 1d and 3b showed that the fiber morphology of the porous Si NFs was maintained by interconnecting with Si crystals having various sizes of pores. By dissolving the magnesia from the MgO/Si crystal composite NFs, the remaining Si crystals might be expected to have the highly porous structures. The Brunauer-Emmett-Teller (BET) measurements resulted that the specific areas of the SiO_2_ NFs and the MgO/Si NFs were 7.8 and 11.8 m^2^/g, respectively. After removal of MgO crystals, the specific area of the porous Si NFs increased to 60.6 m^2^/g. Based on the BET results in Fig. 2c, the Si NFs contained considerable mesoporous pores with diameters between a few nm and tens-of-nm.

The porous Si NFs were coated with graphene through a PECVD method. As shown in Figs. 1e and 3c, the graphene-coated Si NFs maintained their morphology as well as the pore structures. The presence of graphene layers are visually observed in the TEM image in Fig. 3c (see Additional file 1: Figure S5d). Especially at the edge area of the graphene-covered Si NFs, the graphene layers were clearly observed by an obvious bright contrast. As shown in Fig. 2d, the graphene existence of the PECVD-treated Si NFs was verified by Raman spectroscopy. In general, the peaks for the G-band and D-band of the graphite appear in wavelengths near 1580 and 1350 cm^−1^, respectively. The D-band shows disordered features of graphitic sheets [30].

**Fig. 4:**
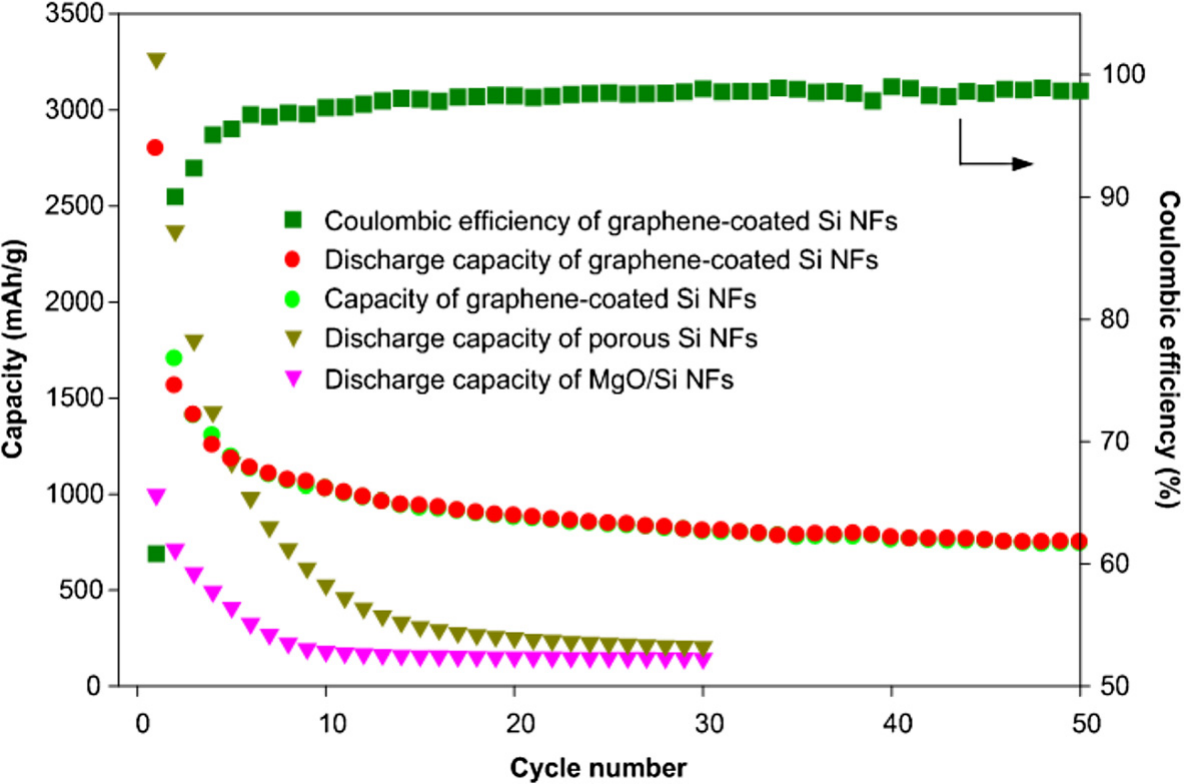
Comparison of specific capacity for the fabricated NFs at 0.5 °C discharging/charging rate and coulombic efficiency of the graphene-coated Si NFs

In order to examine the electrochemical performance of the prepared NF electrodes, the cells assembled using each sample were cycled at a rate of 0.5 C by using a galvanostatic charge/discharge process. As shown in Fig. 4, the battery cell of the MgO/Si NFs materials showed a fast decaying of capacity from 1000 mAh/g. In the case of the Si NFs, the cell reached a high capacity (approximately 3300 mAh/g), but then rapidly decayed, indicating that the Si NFs might experience a volume expansion during the cycling. It could lead to break their structures and disconnect the electron path among the active materials due to the pulverization. It should be noted that the Si NFs underwent a serious stress while being washed to remove the magnesia using the acidic HCl solution, which made them further susceptible to pulverization during the cycling. Meanwhile, the cycling of the graphene-coated Si NFs illustrated a quite different behavior compared to two samples aforementioned. During the initial cycles, the capacity was dropped but retained up to 760 mAh/g after 50 cycles. In the first cycle, a relatively large irreversible capacity was observed by a result of the formation of solid electrolyte interfaces (SEI). The formation of the SEI layer occurred on the surface of the electrodes due to the decomposition of the electrolyte during the initial cycles [31]. In addition, the residual SiOx in the graphene-coated Si NFs might react with Li-ions (SiOx + 2xLi ↔ Si + x Li_2_O) so that the lots of excess Li-ions were consumed during the first discharging and eventually caused an irreversible capacity loss in the initial discharge/charge process [32].

In recent years, several methods to develop Si-based nanoparticles or nanowires using Mg reduction have been reported in order to utilize them as Li-ion batteries [25, 33]. According to the final material composition of the aforementioned researches, their capacities were ranged from 500 to 2000 mAh/g. Despite the existence of SiOx in the fabricated material, the graphene-coated Si NFs also showed comparable cycling performance. The capacity result in this study was in good agreement with a recent report [33]. The capacity of the graphene-coated Si NFs was drastically dropped during a few initial cycles but quickly reached a plateau, indicating that the Si NFs might not be fully covered by graphene. Even so, the structural stability was enhanced by the composite structures of graphene-coated Si NFs.

**Fig. 5:**
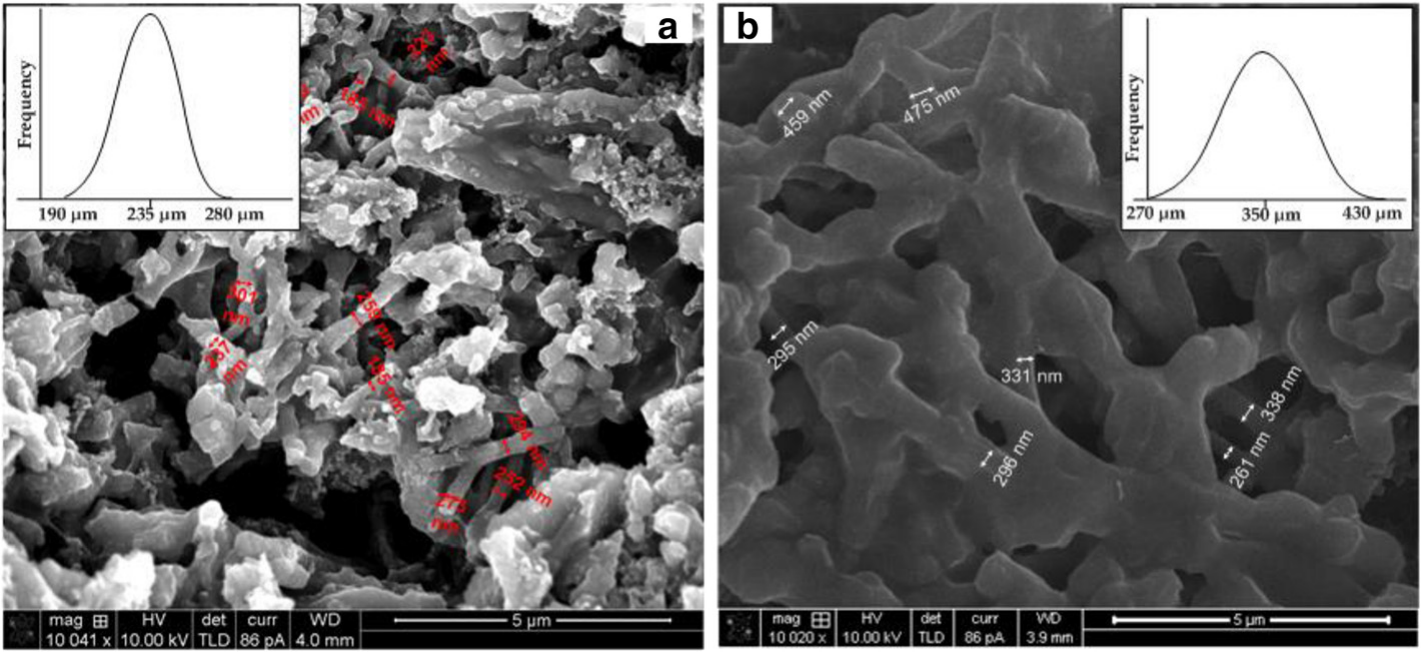
SEM images of disassembled pristine electrode (**a**) and 50-cycled electrode (**b**) (the *insets* represent the distribution of the graphene-coated Si NFs’ diameters)

As can be seen in Fig. 5b, the morphology of the SEI layer was observed on the electrode surface after 50 cycles [34]. The graphene-coated Si NFs electrode after 50 cycles was investigated to compare them with those in the fresh electrode by SEM images (Fig. 5a, b). The graphene-coated Si NFs in the fresh electrode exhibited 190–280 nm in diameter (average 235 nm). Meanwhile, the diameters of the graphene-coated Si NFs after 50 cycles were enlarged up to approximately 150 % when compared to the pre-cycle samples (range of 270–430 nm and average 350 nm in diameter). In this measurement, it was difficult to extract an exact thickness of the fibers from the SEM image so there may be some bias about the measured diameters because of the SEI layer on the surface. However, it was noticeable that the graphene-coated Si NFs showed the continuous fiber structures, suggesting that the fibers were not broken during the cycling. The graphene-coated Si NFs showed an improved cycling performance of a capacity retention compared to the pure Si NFs. Therefore, it might be inferred that the graphene-coated Si NFs might suppress the volume expansion by confining the Si NFs with the graphene layers and could increase the electrical conductivity in their electrode [35]. It might be noted that the SEI formation leads to a difficulty in developing a commercial full cells using Si-based material anodes. Currently, the full cells using Si anodes are our ongoing work to overcome the challenges of the irreversible capacity loss and SEI formation.

## Conclusions

The polymer/Si NFs were successfully fabricated by a simple electrospinning technique with an environmental-friendly spinning dope of water-based solution. By reducing the as-spun fibers with Mg at 650 °C and coating them with graphene through a PECVD process at 400 °C, the final graphene-coated Si NFs were obtained. The fabricated Si NFs revealed continuous fiber shapes with diameters ranging from 190–280 nm. After 50 cycles, the graphene-coated Si NFs did not significantly change their volume. The intermingled and graphene-covered Si NFs showed a high irreversible capacity loss at a few initial cycles, which was attributed by the remaining SiOx in the fabricated materials. After a few cycles, however, they exhibited a stable electrochemical performance that resulted in 760 mAh/g at the 50th cycle. Compared to the Si NFs without the graphene, the graphene-coated Si NFs improved their cycling performance by the confinement of porous-thin Si NFs with graphene. The graphene would suppress the volume change of Si NFs and increase the electric conductivity on the electrode. The approach in this paper proposed that a mass-scale fabrication under an eco-friendly technique would be feasible and the fabricated Si NFs could be utilized in anode application of Li-ion batteries.

## Additional file

Additional file 1:
**This file contains detailed figures to explain the additional informaton of the process and materials.** These include solution images, FTIR, and TEM.

## Abbreviations

EDX: energy dispersive X-ray
Li: rechargeable lithium
Mg: magnesium
NFs: nanofibers
PECVD: plasma-enhanced chemical vapor deposition
SEM: scanning electron microscope
Si: silicon
TEM: transmission electron microscope
XRD: X-ray diffraction
