# Supplementary material for: Facile Synthesis of Porous Silicon Nanofibers by Magnesium Reduction for Application in Lithium Ion Batteries
Source: Nanoscale Res Lett. 2015 Oct 28;10:424. doi: 10.1186/s11671-015-1132-8 (PMC4624685; doi:10.1186/s11671-015-1132-8)
Supplement: Additional file 1: — This file contains detailed figures to explain the additional informaton of the process and materials. These include solution images, FTIR, and TEM. [file 11671_2015_1132_MOESM1_ESM.docx]

**Additional 1: Supplementary figures**

**Facile synthesis of porous silicon nanofibers by magnesium reduction for application in lithium ion batteries**

Daehwan Cho,*^a,^** Moonkyoung Kim,*^b^* Jeonghyun Hwang,*^b^* Jay Hoon Park,*^c^* Yong Lak Joo,*^a^* and Youngjin Jeong, *^d,^**

*^a,^**School of Chemical and Biomolecular Engineering, Cornell University, Ithaca, NY 14853 USA.

^b^School of Electrical and Computer Engineering, Cornell University, Ithaca, NY 14853, USA.

^c^Department of Chemical Engineering, Massachusetts Institute of Technology, Cambridge, MA, 02139, USA.

^d^Department of Organic Materials and Fiber Engineering, Soongsil University, Seoul 156-743, Korea. E-mail: yjeong@ssu.ac.kr (Y. Jeong)


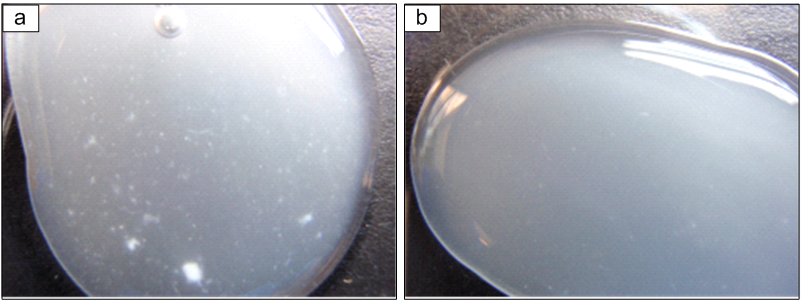


**Figure S1** Digital photos of PVA/Si tetraacetate hybrid solutions prepared by (a) using water solution at room temperature and (b) using water/acetate acid (7/3 wt%) mixture solution in an ice bath.

**Figure S2** Schematic drawing of PECVD process chamber.

**Figure S3** Schematic representation of the reaction between silicon tetraacetate precursor and PVA polymer.

**Figure S4** FTIR spectra of samples: (a) Silicon tetraacetate particles, (b) Pure PVA NFs, (c) PVA/Si tetraacetate hybrid NFs, (d) SiO_2_ NFs, (e) Si/MgO NFs, and (f) Si NFs.


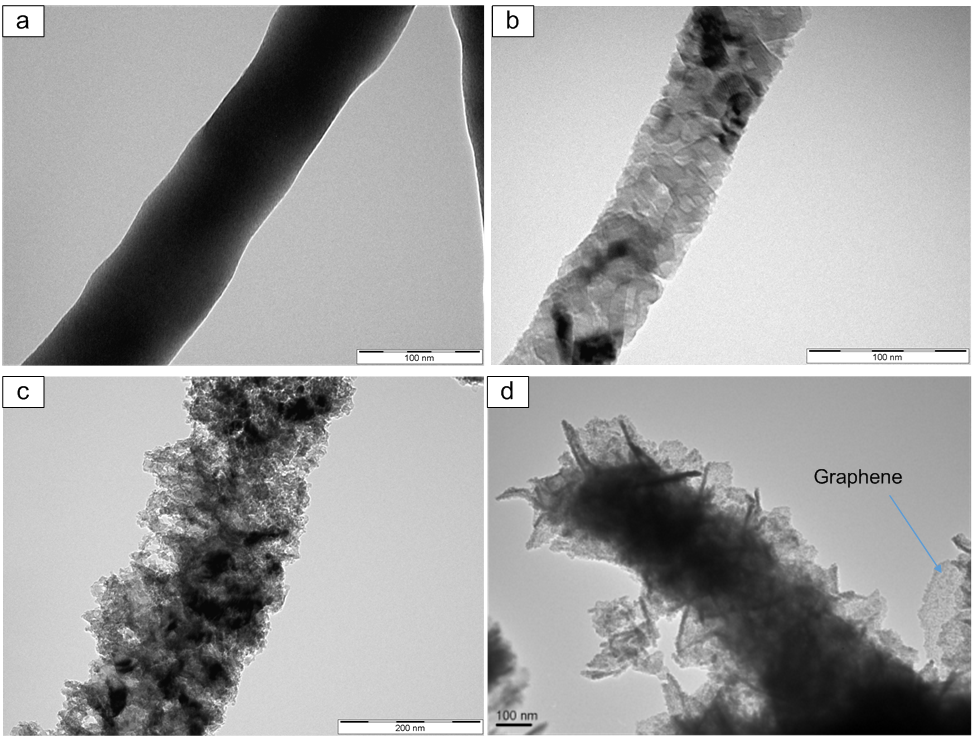


**Figure S5** Enlarged TEM images of the fabricated fibers at each step: (a) SiO_2_ NFs, (b) Si/MgO composite NFs, (c) porous Si NFs, and (d) graphene-coated Si NFs.
